# Natural Selection Drives Codon Usage Bias in the Mitochondrial Genome of *Ligula intestinalis* (Linnaeus, 1758) Gmelin, 1790 (Cestoda: Diphyllobothriidea): Insights from Comparative Genomics and Optimal Codon Identification

**DOI:** 10.3390/ani16172723

**Published:** 2026-09-01

**Authors:** Shun-Jie Wang, Shi-Min Zeng, Feng-Cheng Liu, Jin-Nan Hu, Shun-Hai Wang, Li Zhang, Cheng Yue, Cui-Lan Hao

**Affiliations:** 1 College of Veterinary Medicine, Xinjiang Agricultural University, Urumqi 830052, China; wangshunjie315010@outlook.com (S.-J.W.); qisili740z@163.com (S.-M.Z.); lfc2004@126.com (F.-C.L.); hujn121@outlook.com (J.-N.H.); woesh_1006@163.com (S.-H.W.); lilizhang21@126.com (L.Z.); yuechengxnd@aliyun.com (C.Y.); 2 Xinjiang Regional Key Laboratory of Clinical Veterinary Medicine Research, Urumqi 830052, China; 3 Xinjiang Key Laboratory of New Drug Research and Development for Herbivorous Animals, Urumqi 830052, China

**Keywords:** *Ligula intestinalis*, Diphyllobothriidea, mitochondrial genome, codon usage bias, phylogenetic analysis, natural selection

## Abstract

Codon usage bias (CUB) is a useful indicator of evolutionary forces shaping mitochondrial genomes. Codon usage bias in mitochondrial genomes of Diphyllobothriidae and especially in *Ligula intestinalis* was characterized. The roles of natural selection and mutation pressure in framing this bias were evaluated on the basis of 12 protein-coding genes in Diphyllobothriidae. The complete mitogenome (13,725 bp) of *L. intestinalis* comprises 12 protein-coding genes (PCGs), 22 tRNAs, and two rRNAs, all positioned on the heavy strand, and contains an overall AT content of 66.15%. The mean CAI (0.176), CBI (−0.105), and ENC (45.33) and an evident preference for U-ending codons observed in all examined genes indicate weak CUB. Neutrality, ENC, and PR2 plots consistently demonstrate that natural selection is the predominant force driving CUB and contributes approximately 56% in *L. intestinalis* and 83% in other Diphyllobothriidea species, with mutation pressure playing a secondary role. Phylogenetic reconstruction supported the monophyly of Diphyllobothriidea, confirmed the paraphyly of *Diphyllobothrium* as traditionally defined, and placed *Ligula* and *Digramma* as sister taxa. These findings clarify the evolutionary constraints governing codon usage in cestode mitogenomes and provide practical resources for codon optimization in heterologous gene expression and genetic studies of this economically important parasite.

## Simple Summary

The codon usage bias of mitochondrial genomes in Diphyllobothriidae and especially in *Ligula intestinalis* was characterized. The functions of natural selection and mutation pressure in framing this bias were evaluated on basis of 12 protein-coding genes (PCGs) in Diphyllobothriidae. The weak codon usage bias preferred A- or C-ending codons in the PCGs of Diphyllobothriidae under primary drive by natural selection. Phylogenetic reconstruction supported the monophyly of Diphyllobothriidea. These data help clarify cestode mitochondrial evolution and underscore that natural selection dominantly structures the genetic architecture of Diphyllobothriidea.

## 1. Introduction

Mitochondrial genomes are particularly useful for evolutionary biology, population genetics and phylogenetic reconstruction [1,2,3]. The mitochondrial genomes of metazoans contain 37 proteins, 22 tRNAs, and two rRNAs [3]. Parasitic flatworms (Platyhelminthes: Neodermata) have a different code in mitochondrial genomes, which lack *atp8* in most species and have large AT bias [4,5]. These features have received increasing attention because they may reflect an adaptive response to parasitic lifestyle and provide valuable phylogenetic markers [6].

*Ligula intestinalis* (Linnaeus, 1758) Gmelin, 1790 (Cestoda: Diphyllobothriidea) is a common cestode parasite that attacks freshwater fishes as intermediate hosts and piscivorous birds as final hosts [7,8,9,10]. This parasite has significant ecological and economic effects and often causes host growth degradation, castration of reproductive eggs, and population decline in affected fish communities. In the last 20 years, the mitochondrial genomes of several *Ligula* isolates have been studied, which reveals a conserved architecture of 13.6–13.7 kb of 12 protein-coding genes (PCGs), 22 tRNAs and 2 rRNAs [11,12,13]. A recent study on a Neotropical lineage from Lake Titicaca reported an A + T content of 66.32% and a pronounced regional variation in genes *atp6* [1,2], *nad5* and *nad6* [14]. Comparative mitogenomes have also suggested the synonymy of *Ligula* and *Digramma* [15].

Codon usage bias (CUB) universally occurs across all life domains because mutation pressure is induced by natural selection [16,17,18]. In mitochondrial genomes, codon usage patterns are very interesting because they reflect mutational constraints by replication and transcription machinery and selective pressures due to translation efficiency [18]. In parasitic flatworms, a different mitochondrial code existed in the 1990s (AUA = isoleucine, AAA = asparagine, UGA = tryptophan, UAA = tryptophan, etc.) [5,19,20]. Base composition and codon usage variation are quite large among neodermatans. The GC content at codon position 3 (GC3) is remarkably less than GC1 and GC2 due to mutation bias towards AT [21,22,23,24].

Despite these advances, we have not yet been able to study the codon usage patterns of Diphyllobothriidae cestodes. Most studies focus on taeniid and hymenolepidid cestodes that have medical and veterinary importance, such as Echinococcus and Taenia [25,26]. For *L. intestinalis*, although complete mitochondrial genomes have been obtained from Asian and South American populations [14,15], its CUB is not systematically analyzed [1,2,3]. In addition, to what extent codon usage patterns vary among different lineages and whether the variation is due to local adaptation to different host environments are unknown [27,28,29].

This study is aimed to provide a full examination of CUB in the mitochondrial genome of *L. intestinalis* isolated from *Gobio acutipinnatus* in the Irtysh River, China, and to extend this investigation to a broader comparative framework encompassing 21 Diphyllobothriidea species. This aim is achieved using composition, correlation, and correspondence analyses; neutrality, ENC, and PR2 plots; and optimal codon identification. This study will help clarify the evolutionary forces shaping the mitochondrial genomes of parasitic flatworms and offer valuable resources for upcoming studies on population genetics and the phylogeography of this economically important parasite [30].

## 2. Materials and Methods

### 2.1. Sample Origin and DNA Preparation

*L. intestinalis* was sampled in the Habahe section of the Irtysh River (coordinates referenced from Zhang et al.) in May 2023. The samples of *L. intestinalis* were characterized morphologically, kept in 95% ethanol, and subsequently stored at −20 °C. Total genomic DNA of *L. intestinalis* was obtained with an EasyPure^®^ Genomic DNA kit as per the manufacturer’s introduction. DNA quality was tested via 1% agarose gel electrophoresis, and high-quality DNA samples were sequenced by Personalbio Biotechnology Co., Ltd. (Nanjing, China) on Illumina [31].

### 2.2. Genome Assembly

A paired-end library with an insert size of 400 bp was built with the whole-genome shotgun plan and sequenced on Illumina NovaSeq. The quality of primary sequencing data was checked with FastQC v0.11.9 (http://bioinformatics.babraham.ac.uk/projects/fastqc) (accessed on 9 September 2024). Adapter removal and data filtering were performed on AdapterRemoval 2 [32] to trim 3′ adapter contamination. Quality correction of reads based on K-mer distribution was conducted using SOAPec 2.01 [33]. High-quality clean reads were gathered de novo using A5-miseq 20150522 [34] and SPAdes 3.9.0 [35] to generate contigs and scaffolds. The resulting assembly was refined using Pilon 1.18 [36] to obtain the final mitochondrial genome sequence.

### 2.3. Data Retrieval

The complete mitochondrial genome sequences of 21 Diphyllobothriidae species were retrieved from NCBI (https://www.ncbi.nlm.nih.gov/), including one newly sequenced *L. intestinalis* genome generated in this study and 21 publicly available sequences. One additional species was included as an outgroup. The selection criteria required that the mitochondrial genome sequences be publicly available and fully annotated. The relevant information, including accession numbers, organism names, taxonomic affiliations, genome lengths, and references, is summarized in Table 1. The final dataset included two *L. intestinalis* sequences and 20 other diphyllobothriidean taxa for comparative analysis. The mitochondrial genome lengths ranged from 13,575 to 13,947 bp. The complete sets of mitochondrial genes were available for all selected taxa and were used for subsequent comparative genomic and codon-usage analyses.

### 2.4. Genome Structure and Composition Analysis

The complete mitochondrial genome sequences of the 22 Diphyllobothriidae species, including two *L. intestinalis* sequences and 20 other species, were acquired from NCBI. The sizes and nucleotide compositions of the complete mitogenomes, PCGs, tRNAs, and rRNAs were compared. The lengths and AT contents of the three genomic components were calculated, and AT- and GC-skew values of the PCGs were determined as follows: ATskew = (A − T)/(A + T) and GCskew = (G − C)/(G + C). Comparative analyses and box plots were finished on GraphPad Prism 8.0.2 [56].

### 2.5. Circular Genome Map

The annotated mitochondrial genome in the GenBank format was visualized as a circular map using OGDraw v1.1.1 [57], with genes color-coded according to functional categories.

### 2.6. Codon Usage Parameter Calculation

Twelve PCGs were extracted from the mitochondrial genome using a feature extraction tool (www.detaibio.com/sms2/genbank_feat.html) (accessed on 1 October 2025). Codon usage parameters were tested on CodonW 1.4.2 [58] and custom Python scripts v3.13. Overall GC content (GC); GC contents at codon positions 1, 2, 3 and synonymous position 3 (GC1, GC2, GC3, GC3s); mean GC content of positions 1 and 2 (GC12); effective number of codons (ENC); codon adaptation index (CAI) [16]; codon bias index (CBI); frequency of optimal codons (FOP); protein length (L-aa); relative synonymous codon usage (RSCU) [59,60,61] were computed for each PCG. Correlations among the major codon usage indices were analyzed using R scripts.

### 2.7. Neutrality Plot Analysis

The contributions of mutation pressure and natural selection to CUB were evaluated via neutrality plots using GraphPad Prism 8.0.2 [56]. GC12 was drawn against GC3. A slope near 1 implies similar base compositions across the three positions, indicating mutation pressure as a dominant factor. A slope close to 0 indicates divergence in base compositions between positions, suggesting natural selection as a primary factor. The distribution of genes relative to the diagonal was used to determine the relative influence of these two forces [62].

### 2.8. ENC-Plot Analysis

Each PCG was plotted in a two-dimensional coordinate system with GC3s as the x-axis and ENC as the y-axis. A standard curve was created according to the expected ENC as per the null hypothesis of no selection. The frequency distribution of the ENC ratio (ENCRatio) was calculated to further test deviation from the curve. Genes below the curve are considered to be most influenced by natural selection, while those above the curve are more affected by mutation pressure [4].

The standard curve was plotted as follows:

(1) $$\mathrm{ENC}\;=\;\frac{\mathrm{ENCexp}\;-\;\mathrm{ENCobs}}{\mathrm{ENCexp}}$$

(2) $$\mathrm{ENCexp}=2+\mathrm{GC}3s+\frac{29}{{\mathrm{GC}3s}^{2}+{\;(1\;-\;\mathrm{GC}3s)}^{2}}$$

### 2.9. PR2-Plot Analysis

A standard curve was calculated as follows: PR2-plot analysis [63] was conducted using SPSS 25.0 [64] for parity rule violations. For each PCG, A3/(A3 + T3) was drawn against G3/(G3 + C3). The origin (0.5, 0.5) is the theoretical point where A = T and G = C are positioned away from the center. Genes farther from the center are biased codon usage, while those near the center are not significantly biased.

### 2.10. Recognition of Optimal Codons

Of the genes ordered by ENC, the bottom 20% and top 20% were chosen to construct low- and high-expression genesets, respectively. The distance in RSCU (ΔRSCU) between the two sets was calculated as follows:

(3) ∆RSCU = RSCUhigh expression genes − RSCUlow expression genes

Δ*RSCU* = *RSCUhigh* − *RSCUlow*

The optimal codons were identified by combining two criteria: ΔRSCU ≥ 0.08 and high-frequency codons (RSCU > 1.00) across all genes [5,65].

### 2.11. Phylogenetic Analysis

Nucleotide sequences representing 12 mitochondrial PCGs from the 22 Diphyllobothriidae species were retrieved and processed with PhyloSuite v2 [66]. For each PCG, multiple sequence alignment was applied independently on MAFFT 7.313 [67], followed by codon-aware alignment refinement through MACSE v.2.0.3 [68]. Ambiguous alignment sites were excluded with Gblocks 0.91b by applying its default settings [69]. The resulting 12 single-gene alignments were then concatenated into a single supermatrix in a fixed order on PhyloSuite v2. Optimal partitioning strategies and nucleotide substitution models for both Bayesian inference (BI) and maximum likelihood (ML) were determined using ModelFinder v 1.5.4 [70], with the Akaike Information Criterion (AIC) and the corrected AIC (AICc) as the selection criterion, respectively.

In the BI analysis, the four Markov chain Monte Carlo chains were run in MrBayes 3.2.6 [71] for 2,000,000 generations. Trees were collected at 1000-generation intervals, and the initial 25% of samples were abandoned as burn-in. The ML analysis was estimated with IQ-TREE v.3.0.1 [72] by employing 5000 ultrafast bootstrap replicates. All resulting topologies were visualized on the iTOL web platform [73] (https://itol.embl.de/). *Senga ophiocephalina* was designated as the outgroup, and node support was summarized by both Bayesian posterior probabilities and ML bootstrap percentages.

## 3. Results

### 3.1. Structural Features of L. intestinalis Mitochondrial Genome

**Figure 1:**
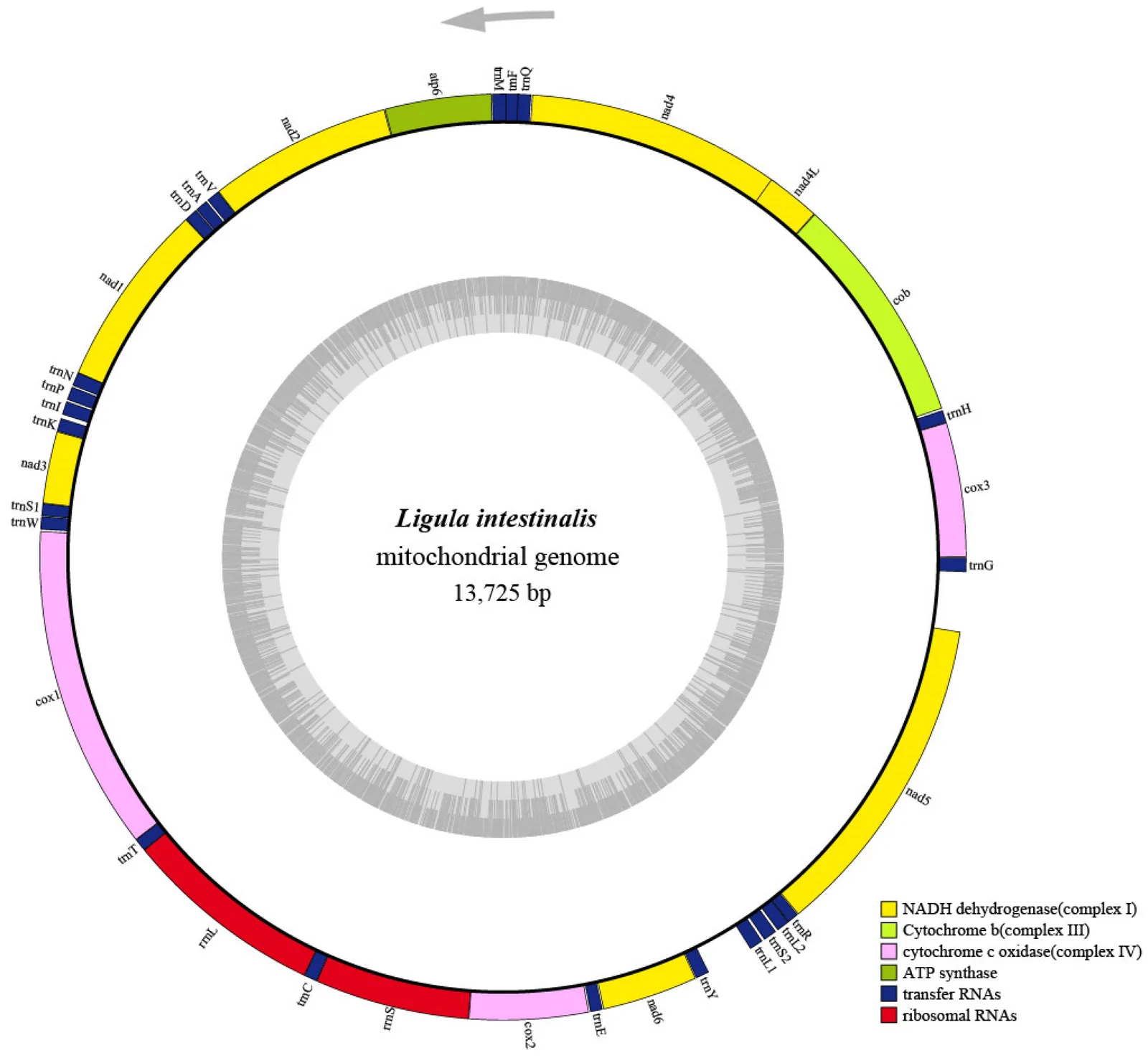
Circular map of the mitochondrial genome of the tapeworm *L. intestinalis*.

In order to elucidate the structural characteristics of the mitochondrial genome of *L. intestinalis* (GenBank accession No.: PZ486068), a parasite infecting *Gobio acutipinnatus* in Habahe, the complete mitochondrial genome of *L. intestinalis* was sequenced, assembled, and annotated. The sequencing library was generated using Illumina NovaSeg 6000 with paired-end 150 bp reads. The insert-size distribution was approximately unimodal with a mean of 353.18 bp, a median of 351 bp, and a standard deviation of 62.41 bp. The sequencing dataset comprised 31,406,977 clean paired-end reads, corresponding to 9.42 × 10^9^ bases. The average sequencing depth across the assembled mitochondrial genome was 4249.031 ×. A total of 185,415 paired-end reads were mapped to the assembled mitogenome. The insert size was 353.18 ± 62.41 bp. It was found to be a typical closed circular molecule of 13,725 bp in length, comprising 36 genes: 12 PCGs, 22 transfer RNA genes, and two ribosomal RNA genes, all positioned on the heavy strand (Figure 1). Intergenic regions and overlaps were observed, with 24 intergenic spacers from 1 to 284 bp and four overlapping regions spanning 1 to 40 bp. The 12 PCGs totaled 10,041 bp, about 73.16% of the complete genome. The nucleotide composition was 21.41% A, 44.74% T, 13.21% C, and 20.64% G. Of the PCGs, *nad5* was the longest (1569 bp) and *nad4L* the shortest (261 bp). Except *cox3* starting with GTG, all other PCGs began with ATG. Incomplete stop codons (T) existed in *cox3*, *nad3*, and *cox1*, while 9 PCGs had complete stop codons (TAA or TAG) (Table 2). These results suggest that the mitochondrial genome of *L. intestinalis* has a compact organization with interesting codon usage and gene arrangement similar to flatworm genomes.

### 3.2. Analysis of Codon Composition

To test the CUB in the mitochondrial genome of *L. intestinalis*, a parasite infecting *Gobio acutipinnatus* in the Irtysh River, the codon usage patterns of its 12 PCGs were explored (Table 3). The GC1, GC2, and GC3 ranged from 27.73% to 45.79%, 22.99% to 37.48%, and 17.24% to 34.21%, with mean values of 35.37%, 33.79%, and 29.06%, respectively, and GC3 was significantly lower than GC1 and GC2. The average GC3s of the 12 PCGs was 27.02%, with the lowest value observed in *nad4L* (13.40%) and the highest value in *cox2* (32.20%). The GC content of the PCGs changed from 24.52% to 37.37%, with an average of 32.74%, indicating a relatively high AT content in the mitochondrial genome (Table 3). Further analysis revealed that the average CAI of the PCGs was 0.18, with a maximum in *cox2* (0.23) and a minimum in *nad4L* (0.12). The average CBI was −0.105, ranging from −0.195 to −0.043. The ENC ranged from 33.88 to 55.30, with an average of 45.33, and the FOP varied from 0.244 to 0.372, averaging 0.326. Collectively, these results implied that the mitochondrial genome of *L. intestinalis* had weak CUB and low codon adaptation. The correlations between codon usage parameters were analyzed on the major codon usage indexes of the mitochondrial PCGs from *L. intestinalis* (Figure S1). The following pairs of parameters showed significant correlations (*p* < 0.05). The GC content had strong positive correlations with GC1, GC3, and GC3s (correlations of 0.69, 0.85, and 0.87 respectively), whereas the correlation between GC3 and GC3s was 1.00 (highly consistent for GC content in different positions and similar base composition). GC1 was positively related to CAI, CBI and FOP (correlations of 0.75, 0.71 and 0.43 respectively), implying that genes with higher GC1 have higher expression and stronger CUB. CAI was strongly related to CBI and FOP (about 0.71 and 0.65 respectively), and the correlation between CBI and FOP was weak (0.31), suggesting consistency between these indices commonly used to determine gene expression or CUB. ENC was moderately positively related to FOP (r = 0.19), and protein length (L-aa) had weak positive correlations to some GC-related and bias-related indexes (GC2 and GC3).

### 3.3. Confirmation of the Optimal Codon

To identify the optimal codons in the mitochondrial genome of *L. intestinalis*, the PCGs were ranked according to their ENCs. The top and bottom 20% of genes were chosen to build high-expression and low-expression gene libraries, respectively. The RSCU and ΔRSCU were then computed for each library (Table 4). A total of 32 codons exhibited ΔRSCU ≥ 0.08 and were designated as highly expressed codons. Among them, 11 codons ended with C, 10 codons with G, five codons with A, and six codons with U. Notably, 28 codons showed ΔRSCU ≥ 0.30, and 21 codons showed ΔRSCU ≥ 0.50. By intersecting the highly expressed codons with the high-frequency codons identified previously (Table 3 and Table 4), five optimal codons were ultimately identified: CAA, CCU, AGU, GAG, and CGU. Among them, one optimal codon ended with A, one codon with G, and three codons ended with U.

### 3.4. Neutral Plotting Analysis

**Figure 2:**
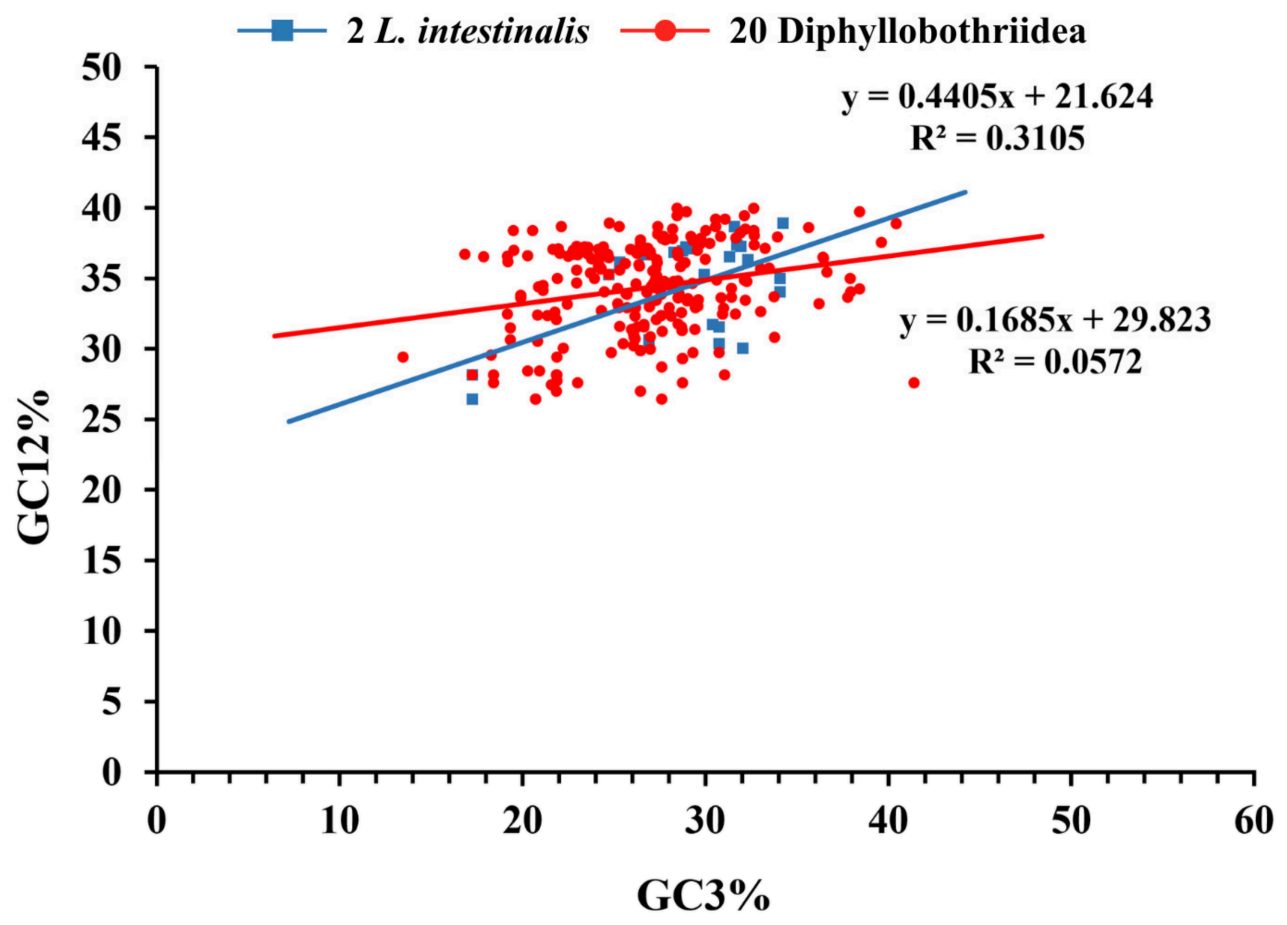
Mitochondrial genome neutral plot of 2 *L. intestinalis* and other Diphyllobothriidae species.

Neutrality plotting on the mitochondrial genome of Diphyllobothriidae was adopted to assess the roles of mutation pressure and natural selection in CUB (Figure 2). The GC3 and GC12 of PCGs ranged from 13.45% to 41.38% and from 26.44% to 40.00%, respectively in the two *L. intestinalis* samples, showing a significant positive correlation (R^2^ = 0.3105, *p* = 0.0043, R^2^ = 0.0572, *p* < 0.001; Figure 2). These consistently high correlations imply that mutational pressure has exerted the main influence on the nucleotide composition of mitogenomes in Diphyllobothriidae over evolutionary time. The regression slopes were 0.4405 for the two *L. intestinalis* samples and 0.1685 for the other Diphyllobothriidae species. Regarding codon usage patterns, the estimated relative contributions of natural selection and mutation pressure in two *L. intestinalis* samples were approximately 56% and 44%, respectively, whereas in the other Diphyllobothriidae species they were 83% and 17%. These results indicated that CUB in Diphyllobothriidea species was framed by both natural selection and mutation pressure, with the former playing the dominant role.

### 3.5. Genome Structure and Composition of Mitogenomes in L. intestinalis and Other Diphyllobothriidae Species

**Figure 3:**
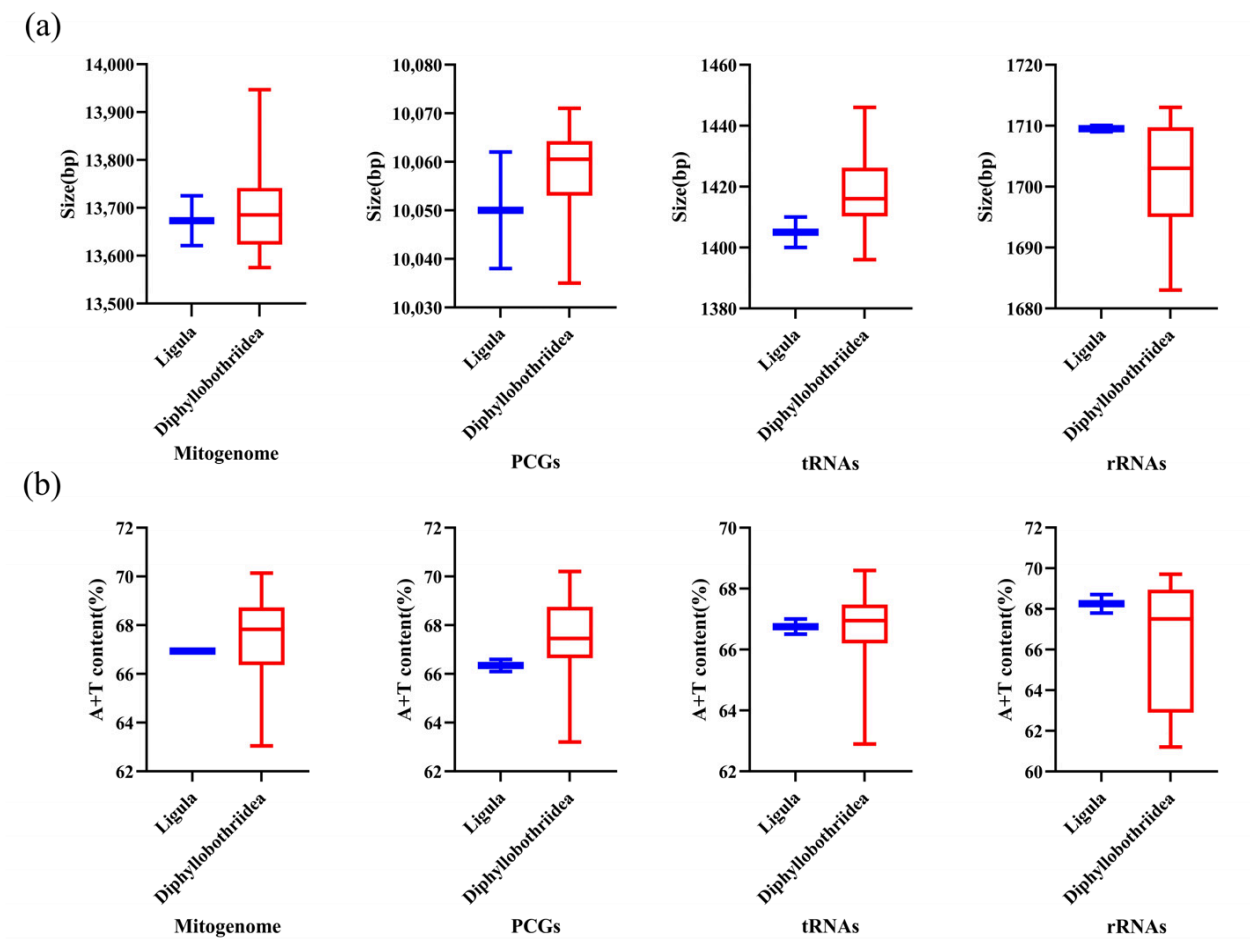
Box plots of sizes (**a**) and A + T content (**b**) of full genome, PCGs, tRNAs, and rRNAs for 2 *L. intestinalis* and 20 Diphyllobothriidea species.

To compare the structural features of Diphyllobothriidae mitogenomes, we examined the sizes and AT contents of PCGs, rRNAs, and tRNAs across 21 species. The PCG lengths fell between 10,038 and 10,062 bp in 2 *L. intestinalis*, the rRNA sequences spanned 1709–1710 bp, and the tRNA sizes ranged from 1400 to 1410 bp (Figure 3a). On contrast, the other Diphyllobothriidae species exhibited broader variability: PCGs varied from 10,035 to 10,071 bp, rRNAs changed from 1683 to 1713 bp, and tRNAs varied from 1396 to 1446 bp. The mean AT-skew and GC-skew values for PCGs were −0.353 ± 0.001 and 0.218 ± 0.002 in 2 *L. intestinalis*, respectively, and were −0.393 ± 0.049 and 0.295 ± 0.057 for the remaining species respectively. Notably, the A + T compositions of PCGs, tRNAs, and rRNAs consistently exceeded 50% in all cases (Figure 3b), indicating a clear AT-rich bias across the three gene categories in Diphyllobothriid mitogenomes.

### 3.6. ENC-Plot Analysis

**Figure 4:**
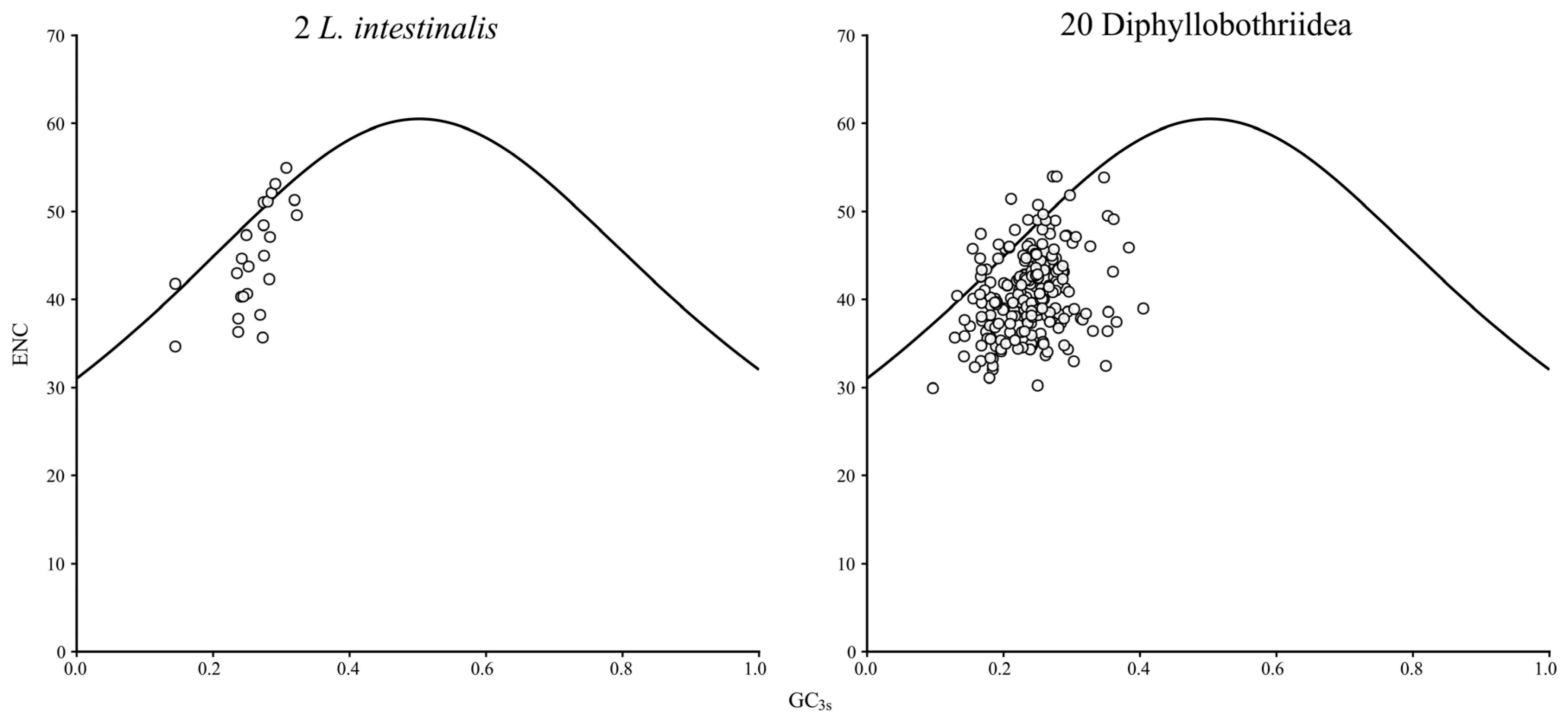
ENC-plot of 12 PCGs in mitochondrial genome of 2 *L. intestinalis* and 20 Diphyllobothriidea species.

To assess the impact of mutation pressure on codon usage patterns in the mitogenomes of Diphyllobothriidea species, we analyzed the link between the ENC and GC3s (Figure 4). In the two *L. intestinalis* samples, ENC ranged from 33.88 to 55.30 and was above 35 in 95.83% of the genes. For the other Diphyllobothriidea species, ENC spanned 29.92 to 53.94 and was greater than 35 in 87.08% of their genes. These findings indicate that CUB is basically weak across the mitogenomes of this order.

The deviation from the standard curve was further assessed by calculating the frequency distribution of the ENCRatio. Among the 24 PCG records from the two *L. intestinalis* mitochondrial genomes, genes with an ENCRatio between −0.05 and 0 accounted for the largest proportion (25.00%) (Table 5). The ENCRatio dominantly fell within −0.05 and 0.20 in the two *L. intestinalis* samples and was mainly distributed between −0.05 and 0.30 in the 20 Diphyllobothriidea species, indicating that only a few genes have very small differences between their ENCexp and ENCobs. Conversely, the CUBs of the majority of genes (with ENC ratios outside this range) were mainly determined by natural selection.

### 3.7. PR2-Plot Analysis

**Figure 5:**
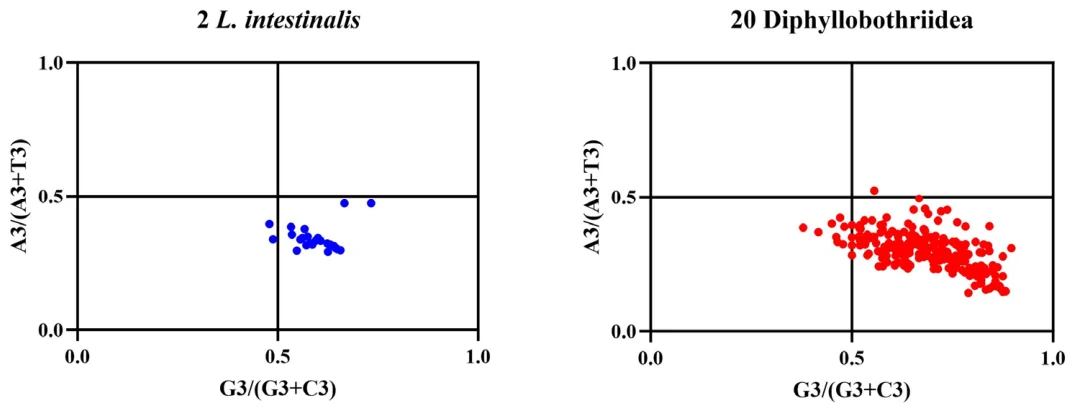
PR2-plot of the mitochondrial genome of 2 *L. intestinalis* and 20 Diphyllobothriidea species.

To assess the codon usage asymmetry at nucleotide position 3 of PCGs in the Diphyllobothriidea mitogenomes, we computed the G3/(G3 + C3) and A3/(A3 + T3) ratios for the 22 species within this order. In the 2 *L. intestinalis,* the numbers of genes assigned to the first, second, third, and fourth quadrants were 0, 0, 2, and 22, respectively (Figure 5). For the remaining 20 Diphyllobothriidea species, the corresponding counts were 1, 0, 8, and 227, respectively (Figure 5). The vast majority of genes across all examined taxa were concentrated in the 4th quadrant, reflecting a consistent preference of T over A and G over C at codon position 3. In all, these findings imply at the mitochondrial codon third site, nucleotides G and T are more commonly employed than A and C in Diphyllobothriidea.

### 3.8. RSCU in 21 Diphyllobothriidea Species

**Figure 6:**
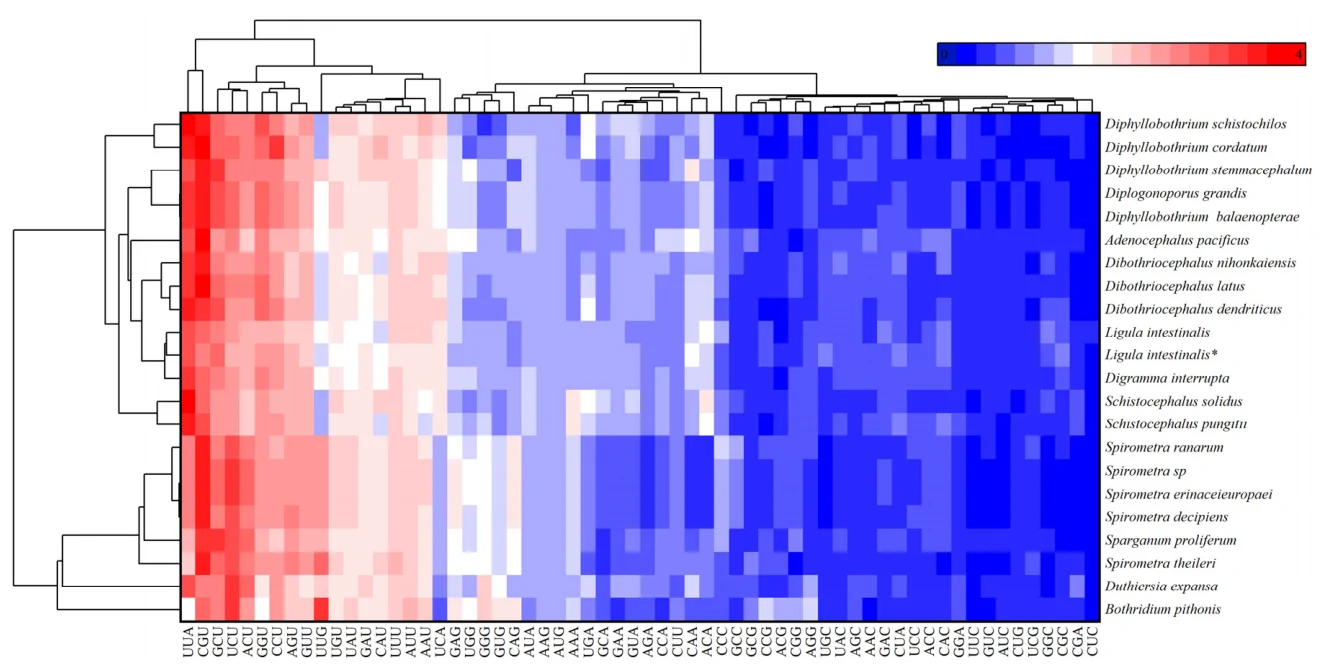
Hierarchical clustering and heat map of RSCU for 21 Diphyllobothriidea species, with a color variation from blue to red in the heat map indicating an increase in RSCU. *: This study.

To elucidate the codon usage patterns within the order Diphyllobothriidea, the RSCUs were calculated from the mitochondrial PCGs of 22 representative species and visualized as a heatmap (Figure 6). The heatmap revealed a pronounced and conserved bias toward codons terminating in U or A across most species. Codons such as UGU, GAU, UUU, GGU, CAU, AUU, AAU, CCU, CGU, UCU, AGU, ACU, UAU, UUA, GUU, and GCU consistently exhibited RSCU > 1.00, while codons ending in C or G, included GCC, GCG, UGC, GAC, UUC, GGC, GGA, CAC, AUC, CUC, CUA, CUG, AAC, CGC, CGA, CGG, UCC, UCG, AGC, AGA, ACC, ACG, GUC and UAC. This pattern aligns with the AT-rich compositional bias characteristic of cestode mitogenomes. Despite this overall conservation, notable interspecific variations were observed. For example, the codons CGU and UUA, which were avoided in the majority of species, displayed RSCU exceeding 1.00 in several lineages, including *L. intestinalis* and two closely related Diphyllobothriidea species, indicating lineage-specific deviations. Similarly, the codon UGC exhibited RSCU > 1 in a small subset of species, but remained below 1 in the remaining taxa. Furthermore, certain codons such as GGU and UCA showed differential usage among species, as some lineages preferred them while others avoided them. The heatmap also revealed that species within the genus Diphyllobothrium clustered together, sharing similar RSCU profiles, whereas *Ligula* and *Spirometra* species formed distinct clusters, suggesting that phylogenetic relatedness partly shapes codon usage preferences. Overall, the results indicate that while a common bias toward U/A-ending codons prevails across Diphyllobothriidea, substantial heterogeneity exists among species, likely driven by differences in mutational pressures, translational selection, or host-specific adaptation.

### 3.9. Phylogenetic Associations

**Figure 7:**
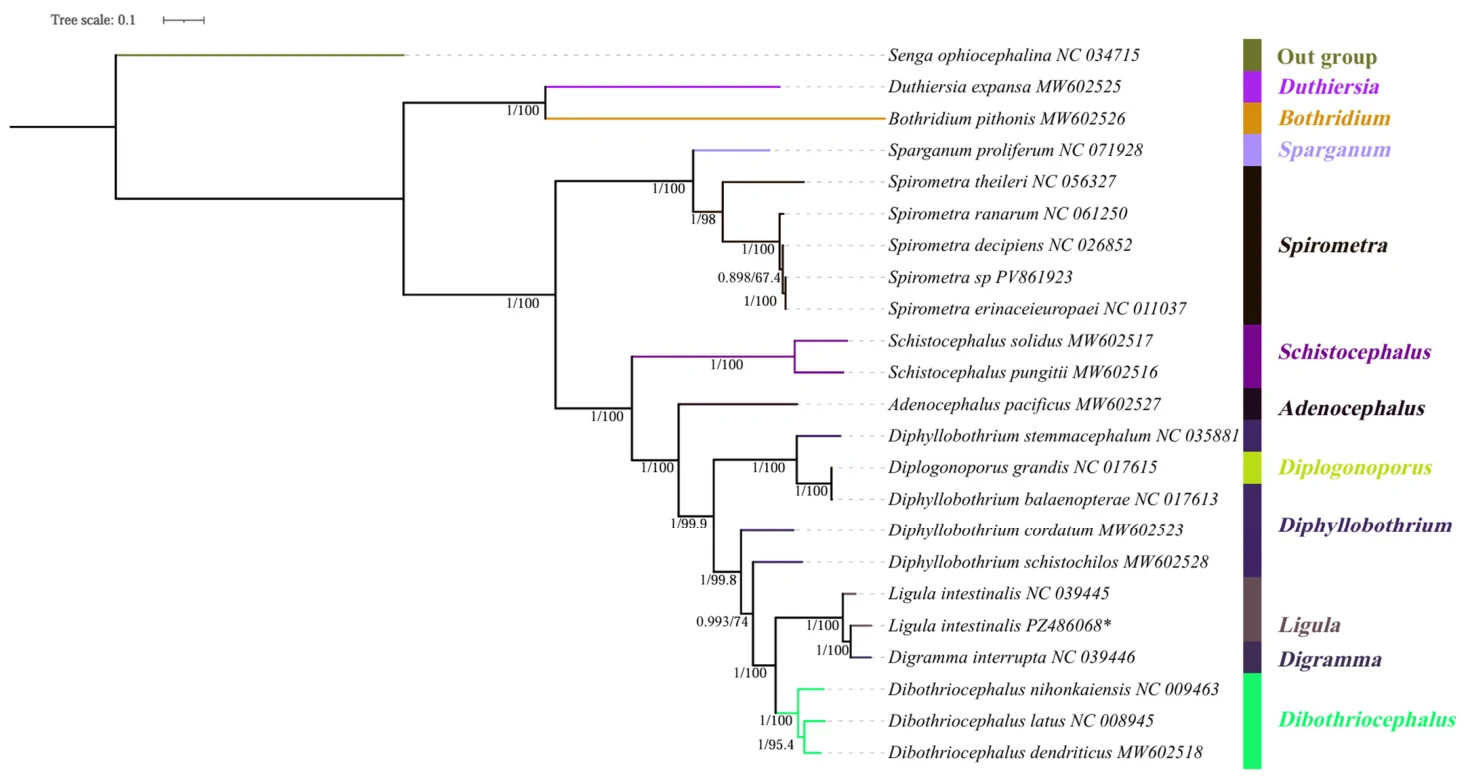
Phylogenetic tree of 12 PCGs from the mitogenomes of Diphyllobothriidea on basis of BI and ML analyses. *: This study.

To infer the phylogenetic relationships among the 21 Diphyllobothriidea species, BI and ML trees were built as per the nucleotide sequences of mitochondrial PCGs, with posterior probability values/bootstrap support indicated at each node (Figure 7). The resulting topology revealed a well-supported monophyletic Diphyllobothriidea clade (posterior probability = 1.00), with the *Senga ophiocephalina* forming the basal outgroup. Within the ingroup, the trees bifurcated into two major clades. Clade one consisted of *Duthiersia expansa* and *Bothridium pithonis*, forming a robust clade (PP = 1.00). Clade two bifurcated into two major subclades: the first subclade comprised *Sparganum proliferum* and all *Spirometra* species (*S. erinaceieuropaei*, *S. decipiens*, *S. ranarum*, *S. theileri*, and *Spirometra* sp.), forming a robust clade (PP = 1.00), with *S. proliferum* positioned as sister to the remaining *Spirometra* members. The second major subclade contained the remaining genera and was further subdivided into two lineages. One lineage united *Schistocephalus* (*S. pungitii* and *S. solidus*) with *Adenocephalus pacificus* and *Diplogonoporus grandis* (PP = 1.00). The other lineage grouped all *Diphyllobothrium* species (*D. schistochilos*, *D. cordatum*, *D. balaenopterae*, *D. stemmacephalum*) with *L. intestinalis* (two accessions), *Digramma interrupta*, and the two *Dibothriocephalus* species (*D. dendriticus* and *D. latus*). Within this latter group, *Ligula* and *Digramma* formed a strongly supported clade (PP = 1.00), which is consistent with their close taxonomic affinity. Notably, the genus *Diphyllobothrium* appeared as a paraphyletic assemblage, as *D. balaenopterae* branched separately from the other three species, which formed a subclade with high support. The tree further indicated that *Dibothriocephalus* was sister to the *Ligula-Digramma-Diphyllobothrium* complex, while *Diplogonoporus* and *Adenocephalus* occupied an intermediate position between the *Schistocephalus* clade and the *Diphyllobothrium*-related clade. All internal nodes received bootstrap values of 0.89 or higher, with the majority at 1.00, indicating high statistical confidence. Overall, the phylogenetic reconstruction robustly delineates the intergeneric relationships within Diphyllobothriidea, confirming the polyphyly of *Diphyllobothrium* as traditionally defined and supporting the distinctiveness of *Ligula* and *Digramma* as sister taxa. These results provide a solid framework for interpreting the codon usage patterns found across these species in the context of their evolutionary history.

## 4. Discussion

This work offers a profound characterization of CUB in the mitochondrial genome of *L. intestinalis* from the Irtysh River as well as comparative analyses across 21 Diphyllobothriidea species. The mitogenome of *L. intestinalis* (13,725 bp) exhibits a genome organization (12 PCGs, 22 tRNAs, and two rRNAs on the heavy strand) consistent with previously characterized mitogenomes of Diphyllobothriidea, including a Neotropical lineage from Lake Titicaca (13,657 bp) and Asian isolates (13,621–13,685 bp) [14]. The RSCU analysis revealed an evident preference for U-ending codons across Diphyllobothriidea species, with 60% of overrepresented codons terminating in U, but the overall CUB was weak, as indicated by the mean ENC (45.26) and CAI (0.176). Neutrality, ENC, and PR2 plots consistently revealed that natural selection was the predominant force framing CUB, and mutation pressure played a secondary role. Phylogenetic assays based on the 12 PCGs showed that within the context of the sampled taxa, Diphyllobothriidea formed a well-supported monophyletic clade, whereas the genus *Diphyllobothrium* appeared as a paraphyletic assemblage, with *Ligula* and *Digramma* recovered as sister taxa.

A pronounced AT bias (66.15%) was observed in the mitogenome of *L. intestinalis*, with nucleotide compositions of 21.41% A, 44.74% T, 13.21% C, and 20.64% G. This result accords with the AT-richness of mitochondrial genomes in flatworms, which is generally attributed to elevated mutational pressures favoring AT nucleotides [4,22]. The total GC content of PCGs averaged 32.74%, which is comparable to the 33.7% reported in a previous *L. intestinalis* isolate and the 33.68% observed in the Neotropical lineage [14]. Across the 21 Diphyllobothriidea species examined, the mean AT- and GC-skew values for PCGs were −0.353 ± 0.001 and 0.218 ± 0.002 in the two *L. intestinalis* specimens, respectively, whereas the remaining species exhibited more variable values (−0.393 ± 0.049 and 0.295 ± 0.057). The A + T composition of PCGs, rRNAs, and tRNAs consistently exceeded 50% in all cases, confirming a clear AT-rich bias across all three gene categories in Diphyllobothriid mitogenomes. Notably, GC3 (29.06%) was significantly lower than GC1 (35.37%) and GC2 (33.79%). This pattern is commonly observed in mitochondrial genomes where codon position 3 is less constrained owing to the redundancy of the genetic code [74,75,76]. This composition bias was further reflected in codon usage: a total of 60% of overrepresented codons (RSCU > 1.00) terminated with U, and none terminated with C, implying a strong preference for U-ending codons. Such terminal nucleotide bias is characteristic of the mitochondrial genomes in flatworms and has been attributed to directional mutation pressure during replication [4,77,78,79].

Multiple approaches reach the conclusion that natural selection is the major factor framing CUB in the mitogenome of *L. intestinalis*, and mutation pressure plays a secondary role. Neutrality plot analysis of the two *L. intestinalis* specimens revealed a regression slope of 0.439, and the estimated relative contributions of mutation pressure and natural selection were approximately 44% and 56%, respectively. For the other 20 Diphyllobothriidea species, the regression slope was 0.1685, and natural selection contributed approximately 83%, while mutation pressure contributed 17%. This variation across species suggests that while natural selection consistently predominates, its relative strength may differ among lineages. The ENC-plot analysis further supported this interpretation: in the two *L. intestinalis* specimens, as 95.83% of genes exhibited ENC values above 35, and 20.83% of genes fell within the ENCRatio range of −0.05 to 0.05. For the other species, 87.08% of genes had ENC values above 35, but only 11.67% of genes fell within the same narrow ENCRatio range. The majority of genes with an ENCRatio outside this interval are mainly shaped by natural selection. PR2-plot analysis uncovered asymmetric base usage at codon position 3, with most genes concentrated in the fourth quadrant (A < T, G > C), further supporting the role of natural selection [4,80,81].

The leading role of selection over mutation in framing CUB in *L. intestinalis* is similar to that in other parasitic flatworms. Reportedly, 223 flatworms show parasitism, playing an important role in mitogenomic evolution: parasitic Neodermata worms are strongly selectively driven in contrast to free-living flatworms [82]. Moreover, an analysis of mitochondrial ND genes across five platyhelminth classes reveals that the contributions of natural selection and mutational pressure vary among classes and genes [22]. Another recent study on endosymbiotic rhabdocoel flatworms shows that symbiotic lifestyles have relaxed selection on mitochondrial PCGs and a higher AT content [83]. These results also show quantitatively that even though weak codon bias is strongly biased in the general sense, natural selection is still the main force controlling codon usage behavior in a diphyllobothriidean cestode.

The heatmap analysis of RSCUs across 21 Diphyllobothriidea species revealed that species within the genus *Diphyllobothrium* clustered together, sharing similar RSCU profiles, whereas *Ligula* and *Spirometra* species formed distinct clusters. This phylogenetic clustering suggests that codon usage preferences are partly constrained by evolutionary history, which is consistent with the observation that closely linked species tend to exhibit identical codon usage patterns owing to shared mutational biases and selective regimes [4,80]. However, notable interspecific variations were also observed, indicating that lineage-specific factors, such as differences in host environments or life history traits, may modulate codon usage patterns even among closely related taxa.

The phylogenetic tree reconstructed from the 12 PCGs of 21 Diphyllobothriidea species using both BI and ML methods revealed a well-supported monophyletic Diphyllobothriidea clade (posterior probability = 1.00), with *Senga ophiocephalina* as the basal outgroup. Within the ingroup, *Duthiersia expansa* and *Bothridium pithonis* formed the earliest diverging clade, followed by a major split separating the *Spirometra*–*Sparganum* clade from a lineage containing *Schistocephalus*, *Adenocephalus*, *Diplogonoporus*, *Diphyllobothrium*, *Ligula*, *Digramma*, and *Dibothriocephalus*. Notably, the genus *Diphyllobothrium* appeared as a paraphyletic assemblage, with *D. balaenopterae* branching separately from the other three species, which formed a subclade with high support. The *Ligula* and *Digramma* formed a strongly supported clade (PP = 1.00), which is consistent with their close taxonomic affinity and supports the congeneric status of these two genera [15]. The topology further indicates that *Dibothriocephalus* is sister to the *Ligula–Digramma–Diphyllobothrium* complex, while *Diplogonoporus* and *Adenocephalus* occupy an intermediate position between the *Schistocephalus* clade and the *Diphyllobothrium*-related clade. All internal nodes received bootstrap values of 0.89 or higher, with the majority at 1.00, indicating high statistical confidence. This phylogenetic framework provides a robust basis for interpreting the codon usage patterns observed across these species in the context of their evolutionary history.

## 5. Conclusions

The CUB in the mitochondrial genome of *L. intestinalis* from the Irtysh River was comprehensively analyzed with additional comparative assessments across 21 Diphyllobothriidea species. The mitogenome of *L. intestinalis* was a closed circular molecule of 13,725 bp, containing 12 PCGs, 2 rRNAs, and 22 tRNAs. The natural selection was the dominant force framing codon usage patterns in Diphyllobothriidea, and mutation pressure played a secondary role across lineages. Phylogenetic reconstruction supported the monophyly of Diphyllobothriidea. These findings construct a basis for clarifying the evolutionary dynamics of codon usage in PCGs of cestode mitogenomes.

## Figures and Tables

**Table 1:** GenBank accession No., species, length and reference of mitogenomes in Diphyllobothriidae.

	ID	Organism	Genus	Length (bp)	Reference
1	MW602527.1	*Adenocephalus pacificus*	*Adenocephalus*	13,747	[37]
2	MW602526.1	*Bothridium pithonis*	*Bothridium*	13,756	[38]
3	MW602518.1	*Dibothriocephalus dendriticus*	*Dibothriocephalus*	13,589	[39]
4	NC_008945.1	*Dibothriocephalus latus*	*Dibothriocephalus*	13,715	[40]
5	NC_009463.1	*Dibothriocephalus nihonkaiensis*	*Dibothriocephalus*	13,577	[41]
6	NC_039446.1	*Digramma interrupta*	*Digramma*	13,575	[42]
7	MW602523.1	*Diphyllobothrium cordatum*	*Diphyllobothrium*	13,623	[43]
8	MW602528.1	*Diphyllobothrium schistochilos*	*Diphyllobothrium*	13,608	[44]
9	NC_017613.1	*Diphyllobothrium balaenopterae*	*Diphyllobothrium*	13,720	[45]
10	NC_035881.1	*Diphyllobothrium stemmacephalum*	*Diphyllobothrium*	13,724	[46]
11	NC_017615.1	*Diplogonoporus grandis*	*Diplogonoporus*	13,685	[45]
12	MW602525.1	*Duthiersia expansa*	*Duthiersia*	13,805	[47]
13	NC_039445.1	*Ligula intestinalis*	*Ligula*	13,608	[48]
14	PZ486068	*Ligula intestinalis*	*Ligula*	13,725	This study
15	MW602516.1	*Schistocephalus pungitii*	*Schistocephalus*	13,657	[49]
16	MW602517.1	*Schistocephalus solidus*	*Schistocephalus*	13,621	[50]
17	NC_071928.1	*Sparganum proliferum*	*Sparganum*	13,947	[51]
18	NC_011037.1	*Spirometra erinaceieuropaei*	*Spirometra*	13,607	[52]
19	NC_026852.1	*Spirometra decipiens*	*Spirometra*	13,716	[53]
20	NC_056327.1	*Spirometra theileri*	*Spirometra*	13,725	[54]
21	NC_061250.1	*Spirometra ranarum*	*Spirometra*	13,584	[55]
22	PV8619231	*Spirometra* sp.	*Spirometra*	13,947	[54]
23	NC_034715	*Senga ophiocephalina*	*Senga*	13,816	Out group

**Table 2:** Characteristics of the mitochondrial genes of *L. intestinalis*.

Gene	Location (bp)	Size (bp)	Start Codon	Stop Codon	Intergenic Nucleotides	Anticodon	Direction
*cox3*	1–643	643	GTG	T--	3		+
*trnH*	644–710	67			0	GTG	+
*cob*	714–1820	1107	ATG	TAA	3		+
*nad4L*	1822–2082	261	ATG	TAA	1		+
*nad4*	2043–3293	1251	ATG	TAG	−40		+
*trnQ*	3293–3357	65			−1	TTG	+
*trnF*	3353–3419	67			−6	GAA	+
*trnM*	3416–3482	67			−4	CAT	+
*atp6*	3486–3995	510	ATG	TAA	3		+
*nad2*	3998–4876	879	ATG	TAG	2		+
*trnV*	4878–4941	64			1	TAC	+
*trnA*	4950–5010	61			8	TGC	+
*trnD*	5014–5077	64			3	GTC	+
*nad1*	5078–5968	891	ATG	TAG	0		+
*trnN*	5968–6033	66			−1	GTT	+
*trnP*	6041–6105	65			7	TGG	+
*trnI*	6114–6178	65			8	GAT	+
*trnK*	6198–6261	64			19	CTT	+
*nad3*	6263–6619	357	ATG	TAG	1		+
*trnS1*	6609–6667	59			−11	GCT	+
*trnW*	6670–6732	63			2	TCA	+
*cox1*	6741–8306	1566	ATG	TAG	8		+
*trnT*	8297–8358	62			−10	TGT	+
*rrnL*	8359–9325	967			0		+
*trnC*	9326–9389	64			0	GCA	+
*rrnS*	9390–10,131	742			0		+
*cox2*	10,132–10,701	570	ATG	TAA	0		+
*trnE*	10,703–10,766	64			1	TTC	+
*nad6*	10,772–11,230	459	ATG	TAG	5		+
*trnY*	11,234–11,298	65			3	GTA	+
*NCR1*	11,299–11,523	225			0		+
*trnL1*	11,524–11,590	67			0	TAG	+
*trnS2*	11,603–11,668	66			12	TGA	+
*trnL2*	11,680–11,743	64			11	TAA	+
*trnR*	11,744–11,798	55			0	ACG	+
*nad5*	11,802–13,370	1569	ATG	TAA	3		+
*NCR2*	13,371–13,654	284			0		+
*trnG*	13,655–13,722	68			0	TCC	+

**Table 3:** Characteristics of the PCGs in *L. intestinalis*.

Gene	GC1 (%)	GC2 (%)	GC3 (%)	GC3s (%)	GC (%)	CAI	CBI	FOP	ENC	L-aa
*cox3*	39.25	34.11	26.64	26.64	33.33	0.19	−0.043	0.360	46.59	210.00
*cob*	39.84	34.96	31.71	29.70	35.50	0.21	−0.072	0.350	44.64	358.00
*nad4L*	33.33	22.99	17.24	13.40	24.52	0.12	−0.172	0.244	33.88	86.00
*nad4*	33.81	36.21	34.05	31.60	34.69	0.16	−0.141	0.306	50.76	414.00
*atp6*	35.29	35.29	24.71	23.30	31.76	0.20	−0.116	0.313	41.39	167.00
*nad2*	30.03	33.45	30.38	26.80	31.29	0.16	−0.195	0.272	50.15	290.00
*nad1*	35.69	37.37	31.31	28.90	34.79	0.18	−0.109	0.329	47.49	291.00
*nad3*	27.73	32.77	26.89	26.30	29.13	0.15	−0.156	0.291	37.96	114.00
*cox1*	37.16	37.36	28.93	28.80	34.48	0.19	−0.098	0.341	43.66	511.00
*cox2*	45.79	32.11	34.21	32.20	37.37	0.23	−0.050	0.372	55.30	187.00
*nad6*	29.41	31.37	30.72	27.30	30.50	0.17	−0.101	0.322	40.50	150.00
*nad5*	37.09	37.48	31.93	30.00	35.50	0.17	−0.106	0.331	51.69	512.00
Mean	35.37	33.79	29.06	27.02	32.74	0.18	−0.105	0.326	45.33	278.58

**Table 4:** Relative usage of synonymous codons in the mitochondrial high and low gene expression libraries of *L. intestinalis*.

Amino Acid	Codon	Heigh Expression Gene		Low Expression Gene		ΔRSCU	Amino Acid	Codon	Heigh Expression Gene		Low Expression Gene		ΔRSCU
		Number	RSCU	Number	RSCU				Number	RSCU	Number	RSCU	
Phe	UUU	12	1.60	8	1.78	−0.18	Ala	GCU	2	2.00	1	4.00	−2.00
	UUC	3	0.40	1	0.22	0.18		GCC	0	0.00	0	0.00	0.00
Leu	UUA	8	2.53	12	3.43	−0.90		GCA	1	1.00	0	0.00	1.00
	UUG	3	0.95	2	0.57	0.38		GCG	1	1.00	0	0.00	1.00
	CUU	1	0.32	0	0.00	0.32	Ter	UAA	1	2.00	1	2.00	0.00
	CUC	3	0.95	0	0.00	0.95		UAG	0	0.00	0	0.00	0.00
	CUA	1	0.32	6	1.71	−1.39	His	CAU	4	1.60	1	2.00	−0.40
	CUG	3	0.95	1	0.29	0.66		CAC	1	0.40	0	0	0.40
Ile	AUU	7	1.56	6	1.71	−0.15	Gln	CAA	2	1.00	0	0	1.00
	AUC	2	0.44	1	0.29	0.15		CAG	2	1.00	0	0	1.00
	AUA	5	1.00	3	1.00	0.00	Asn	AAU	3	1.50	3	2.00	−0.50
Met	AUG	5	1.00	3	1.00	0.00		AAC	1	0.50	0	0	0.50
Val	GUU	13	1.93	5	1.82	0.11	Lys	AAA	5	1.00	1	2.00	−1.00
	GUC	2	0.30	1	0.36	−0.06		AAG	5	1.00	0	0	1.00
	GUA	7	1.04	4	1.45	−0.41	Asp	GAU	7	1.56	2	2.00	−0.44
	GUG	5	0.74	1	0.36	0.38		GAC	2	0.44	0	0	0.44
Ser	UCU	4	2.29	3	3.43	−1.14	Glu	GAA	2	0.80	2	1.33	−0.53
	UCC	2	1.14	0	0.00	1.14		GAG	3	1.20	1	0.67	0.53
	UCA	4	2.29	2	2.29	0.00	Cys	UGU	3	1.00	2	2.00	−1.00
	UCG	1	0.57	0	0.00	0.57		UGC	2	1.00	0	0.00	1.00
	AGU	3	1.71	1	1.14	0.57	Trp	UGG	2	1.00	1	2.00	−1.00
	AGC	0	0.00	0	0.00	0.00		UGA	2	1.00	0	0.00	1.00
Pro	CCU	3	1.71	0	0.00	1.71	Arg	CGU	2	2.67	0	0.00	2.67
	CCC	1	0.57	0	0.00	0.57		CGC	0	0.00	0	0.00	0.00
	CCA	0	0.00	0	0.00	0.00		CGA	1	1.33	0	0.00	1.33
	CCG	3	1.71	0	0.00	1.71		CGG	0	0.00	2	4.00	−4.00
Thr	ACU	5	2.50	3	2.4	0.10		AGA	0	0.00	1	1.14	−1.14
	ACC	1	0.50	0	0.00	0.50		AGG	0	0.00	0	0.00	0.00
	ACA	2	1.00	2	1.60	−0.60	Gly	GGU	5	1.33	1	2.00	−0.67
	ACG	0	0.00	0	0.00	0.00		GGC	2	0.53	1	2.00	−1.47
Tyr	UAU	8	1.45	2	2.00	−0.55		GGA	2	0.53	0	0.00	0.53
	UAC	3	0.55	0	0.00	0.55		GGG	6	1.60	0	0.00	1.60

**Table 5:** Frequency distribution of ENCRatio in mitochondrial genome of 2 *L. intestinalis* and 20 Diphyllobothriidea species.

ENC_Ratior_ Range	Number	Frequency
−0.15–−0.10	0/3	0/1.25
−0.10–−0.05	0/3	0/1.25
−0.05–0	6/15	25.00/6.25
0–0.05	3/13	12.50/5.42
0.05–0.10	4/38	16.67/15.83
0.10–0.15	3/50	12.50/20.83
0.15–0.20	4/45	16.67/18.75
0.20–0.25	5/34	12.50/14.17
0.25–0.30	1/27	4.17/11.25
0.30–0.35	0/9	0/3.75
0.35–0.40	0/2	0/0.83
0.40–0.45	0/1	0/0.42

**Note:** Left: 2 *L. intestinalis*; Right: 20 Diphyllobothriidea species.

## Data Availability

The mitochondria genome sequence of *Ligula intestinalis* has been deposited in GenBank of NCBI (https://www.ncbi.nlm.nih.gov/), under the accession number PZ486068.

## Supplementary Materials

The following supporting information can be downloaded at https://www.mdpi.com/article/10.3390/ani16172723/s1, Figure S1: Comparisons between codon main usage bias parameters, * significant correlation (*p* < 0.05).

## Abbreviations

CUB	Codon usage bias
PR2	Parity rule 2
RSCU	Relative synonymous codon usage
ENC	Effective number of codons
PCGs	Protein-coding genes
BI	Bayesian inference
ML	Maximum likelihood
