# Supplementary material for: Natural Selection Drives Codon Usage Bias in the Mitochondrial Genome of Ligula intestinalis (Linnaeus, 1758) Gmelin, 1790 (Cestoda: Diphyllobothriidea): Insights from Comparative Genomics and Optimal Codon Identification
Source: Animals (Basel). 2026 Sep 1;16(17):2723. doi: 10.3390/ani16172723 (PMC13565670; doi:10.3390/ani16172723)
Supplement: Supplementary file 1 [file animals-16-02723-s001.zip › animals-4394448-supplementary.pdf]

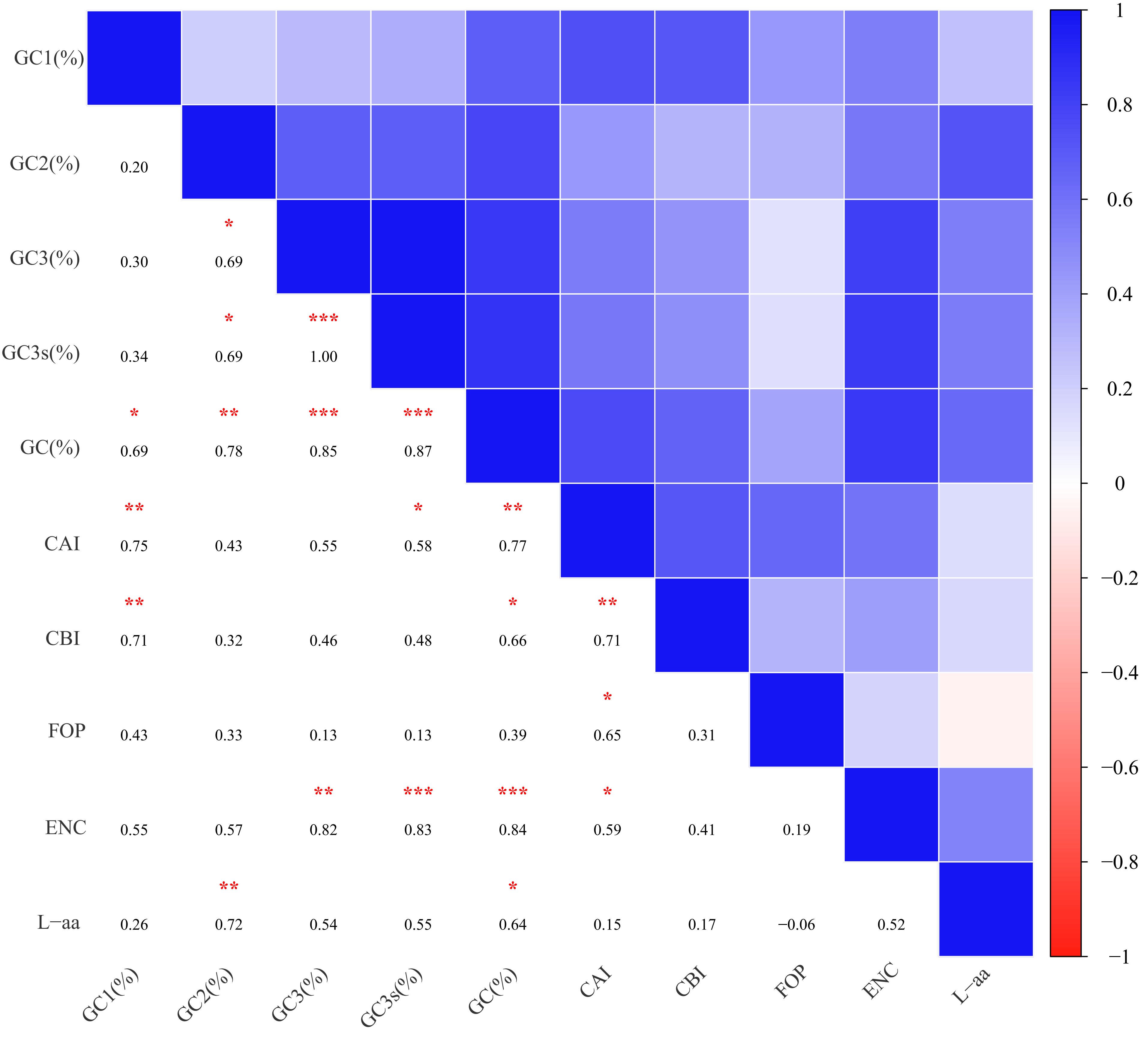
GC1(%)
GC2(%) 0.20
GC3(%) 0.30 0.69 *
GC3s(%) 0.34 0.69 * 1.00 ***
GC(%) 0.69 * 0.78 ** 0.85 *** 0.87 ***
CAI 0.75 ** 0.43 0.55 0.58 * 0.77 **
CBI 0.71 ** 0.32 0.46 0.48 0.66 * 0.71 **
FOP 0.43 0.33 0.13 0.13 0.39 0.65 * 0.31
ENC 0.55 0.57 0.82 ** 0.83 *** 0.84 *** 0.59 * 0.41 0.19
L−aa 0.26 0.72 ** 0.54 0.55 0.64 * 0.15 0.17 −0.06 0.52
GC1(%) GC2(%) GC3(%) GC3s(%) GC(%) CAI CBI FOP ENC L−aa
1
0.8
0.6
0.4
0.2
0
−0.2
−0.4
−0.6
−0.8
−1
